# Acceptance of COVID-19 Vaccine in Pakistan: A Nationwide Cross-Sectional Study

**DOI:** 10.7759/cureus.16603

**Published:** 2021-07-24

**Authors:** Mohammad Aadil Qamar, Omar Irfan, Rubaid A Dhillon, Areesh Bhatti, Mir Ibrahim Sajid, Safia Awan, Wajiha Rizwan, Ali Bin Sarwar Zubairi, Zouina Sarfraz, Javaid Ahmed Khan

**Affiliations:** 1 Internal Medicine, Ziauddin University, Karachi, PAK; 2 Pediatrics, Centre for Global Child Health, Hospital for Sick Children, Toronto, CAN; 3 Internal Medicine, Riphah International University, Rawalpindi, PAK; 4 Pediatrics, The Aga Khan University, Karachi, PAK; 5 Internal Medicine, The Aga Khan University, Karachi, PAK; 6 Pediatrics, The Children Hospital and Institute of Child Health, Lahore, PAK; 7 Pulmonology, The Aga Khan University, Karachi, PAK; 8 Internal Medicine, Fatima Jinnah Medical University, Lahore, PAK

**Keywords:** covid-19, vaccination, acceptability, pakistan, public health

## Abstract

Introduction

The coronavirus disease 2019 (COVID-19) vaccine is available across various countries worldwide, with public-private partnerships ensuring all individuals are vaccinated through a phased approach. Irrespective of the geographical spread, several myths pertaining to the COVID-19 vaccine have stemmed, ultimately limiting the national administration of vaccines and rollouts. This study assessed the acceptance of the COVID-19 vaccine among the general public in Pakistan.

Methods

A pre-validated questionnaire was administered from January 2021 to February 2021 to assess the public attitude and acceptance of the COVID-19 vaccine. Logistic regression analyses were run to identify factors associated with the acceptance among the population.

Results

A total of 936 responses were elicited, where 15% perceived their risk of being infected at 20-30% with an overall 70% agreeing to be vaccinated if recommended. Multivariate analysis identified higher acceptance in the male gender, healthcare workers, and students. Of all, 66% respondents chose healthcare workers and public officials, whereas 15.6% chose scientific literature, and 12.9% chose social media as the most reliable source of COVID-19 information.

Conclusion

Given the relatively greater trust in healthcare providers for information regarding COVID-19, healthcare workers ought to be on the frontline for vaccine campaigns and public outreach efforts, with governmental efforts in addition to the promotion of scientific materials for population-level understanding.

## Introduction

The coronavirus disease 2019 (COVID-19) pandemic began as a cluster of respiratory tract infections of unknown origin in Wuhan, China. As a mutated variant of the coronavirus family and given extensive contact with the center of the outbreak, COVID-19 dispersed worldwide. As of June 29, 2021, 181.5 million confirmed cases have been reported worldwide to the World Health Organization (WHO), with 3.9 million casualties [1,2]. A total of 0.95 million confirmed COVID-19 cases have been reported from Pakistan, as of June 29, 2021 [1,2]. Notably, the vaccine doses administered as of June 29, 2021, are 2.9 billion, as reported to the WHO [1].

Pakistan comprises a diverse population comprising a multitude of ethnic, religious, and socio-economic groups. Among them lies a fraction of the population which has previously opposed the use of polio vaccinations, resulting in Pakistan being one of the three countries that still reports polio cases [3]. Among the many challenges that were faced in the acceptance of polio vaccination programs, one was the alleged myth concerning the biological effects of the vaccine [4]. The myth was propagated primarily by certain religious clerics, which was also raised in the case of COVID-19 vaccines, ranging from the vaccine’s ability to alter DNA to the insertion of microchips in controlling human behaviors [4]. As a low- and middle-income country (LMIC) with poor health infrastructure and a limited budget to provide advanced point-of-care treatment, Pakistan would have had to enforce the mantra of “prevention is better than cure,” to counter the myths [5].

With five vaccines (AstraZeneca, Sinopharm, CanSino, Sputnik V, and Gamaleya) approved for use in Pakistan as of June 29, 2021, along with the phased rollout approach of mass vaccination, this study aimed to assess the acceptance of the COVID-19 vaccine among the general public. This study also aimed to inform medical communities about the causes of gaps in vaccine administration in LMIC, with relevant public health findings to ensure uniform vaccine rollouts.

## Materials and methods

A large-scale, cross-sectional study was conducted to assess the acceptability of the COVID-19 vaccine among the Pakistani population from January 2021 to February 2021. Ethical approval was obtained from the Ethical Review Committee of Islamic International Medical College, Rawalpindi (Riphah/IIMC/ ERC/146). An online questionnaire prepared on Google Forms both in Urdu and English was disseminated to be filled by consenting individuals aged 18 years or more. The study investigators in consultation with subject experts administered a previously validated questionnaire for the study [6,7]. The questionnaire was run through pilot testing consisting of 40 participants comprising various field experts and layman individuals to ensure the clarity and relevance of the contents being assessed. The responses obtained during the pilot testing were not included during the final analysis of data.

The survey consisted of two main sections; first, respondent demographics (socio-economic status and relevant comorbidities), and second, vaccine attitude, to assess vaccine acceptance under different circumstances. The first section incorporated demographic questions with select options addressing age, gender, province, education, employment, income, marital status, occupation, and comorbidity. The second section employed the use of polar questions and the Likert scale on four occasions to assess the level of trust the people placed on the government, reliability of media as a source of accurate pandemic information, compliance to get vaccinated, and to ascertain the severe effects of COVID-19 if the disease was contracted.

The sample size was calculated using OpenEpi (Version 3.01; Open Source Epidemiologic Statistics for Public Health), which was estimated to be 683 participants (95% confidence interval {CI}, bound on the error of 3%, and 50% acceptability). The final enrollment included 1000 participants to account for the accretion rate, and to compensate for any missing data in the final dataset. To analyze the data, SPSS Version 25 (Chicago, IL: IBM® SPSS® Statistics) was utilized. Quantitative variables were represented as mean and standard deviation (SD), whereas frequencies were used for qualitative variables. The comparison of acceptance of vaccines among different groups within the socio-demographic was assessed using the chi-square test and/or Fisher's exact test, where appropriate. Univariate and multivariate analyses were performed to compare the acceptance of the vaccine with the variables of interest. The test result was considered statistically significant if the p-value was 0.05 or less.

## Results

Of the 1000 forms that were distributed, a total of 936 participants completed the online questionnaire, yielding a response rate of 93.6%. A significant majority of the population were females (n=558, 59.6%), aged between 20 years and 29 years (n=411, 43.9%), not-married (n=708, 75.6%) and were residents of the province, Punjab (n=485, 51.8%). A detailed analysis of the patient demographics can be visualized in Table 1.

**Table 1:** Characteristics of the study population (N=936)

Variable	Total: n (%)	Vaccine accepted N=656: n (%)	Vaccine refused N=280: n (%)	p-value
Age (years)				<0.001
18-19	359 (38.4)	226 (34.5)	133 (47.5)	
20-29	411 (43.9)	319 (48.6)	92 (32.9)	
30-39	98 (10.5)	69 (10.5)	29 (10.4)	
40-49	25 (2.7)	19 (2.9)	6 (2.1)	
50-59	28 (3)	16 (2.4)	12 (4.3)	
>60	15 (1.6)	7 (1.1)	8 (2.9)	
Gender				0.009
Male	378 (40.4)	283 (43.1)	95 (33.9)	
Female	558 (59.6)	373 (56.9)	185 (66.1)	
Province				0.01
Sindh	341 (36.4)	261 (39.8)	80 (28.6)	
Punjab	485 (51.8)	318 (48.5)	167 (59.6)	
Balochistan	34 (3.6)	22 (3.4)	12 (4.3)	
Khyber Pakhtunkhwa	64 (6.8)	47 (7.2)	17 (6.1)	
Gilgit-Baltistan	12 (1.3)	8 (1.2)	4 (1.4)	
Education				0.003
No formal education	21 (2.2)	10 (1.5)	11 (3.9)	
Primary school	3 (0.3)	2 (0.3)	1 (0.4)	
Secondary school	105 (11.2)	80 (12.2)	25 (8.9)	
Bachelor/university	608 (65)	441 (67.2)	167 (59.6)	
Master/PhD	185 (19.8)	117 (17.8)	68 (24.3)	
Diploma	14 (1.5)	6 (0.9)	8 (2.9)	
Occupation				<0.001
Unemployed	149 (15.9)	105 (16)	44 (15.7)	
Retired	6 (0.6)	6 (0.9)	0 (0)	
Private sector	187 (20)	147 (22.4)	40 (14.3)	
Government employee	44 (4.7)	38 (5.8)	6 (2.1)	
Business	39 (4.2)	25 (3.8)	14 (5)	
Student	505 (54)	334 (50.9)	171 (61.1)	
Unskilled worker	6 (0.6)	1 (0.2)	5 (1.8)	
Income (in Pakistani rupees)				<0.001
<10,000	52 (5.6)	38 (5.8)	14 (5)	
10,000-49,999	95 (10.1)	71 (10.8)	24 (8.6)	
50,000-100,000	94 (10)	75 (11.4)	19 (6.8)	
>100,000	122 (13)	100 (15.2)	22 (7.9)	
Not applicable	573 (61.2)	372 (56.7)	201 (71.8)	
Marital status				0.005
Married	209 (22.3)	145 (22.1)	64 (22.9)	
Single	708 (75.6)	504 (76.8)	204 (72.9)	
Divorced	19 (2)	7 (1.1)	12 (4.3)	
Occupation				0.003
Healthcare workers	133 (14.2)	107 (16.3)	26 (9.3)	
Medical students	298 (31.8)	216 (32.9)	82 (29.3)	
None of the above	505 (54)	333 (50.8)	172 (61.4)	
Presence of COVID-19 infection in family or friends	427 (45.6)	333 (50.8)	94 (33.6)	<0.001
Comorbidity				0.69
Yes	130 (13.9)	93 (14.2)	37 (13.2)	
No	806 (86.1)	563 (85.8)	243 (86.8)	

Around half of the study population (n=427, 45.6%) reported a relative or a friend who had contracted COVID-19. The participants reported their perceptions toward COVID-19 and response towards the vaccine as a percentage (i.e. 0-10%, 10-20%), shown in Table 2. One-third of the population believed that they had less than a 10% risk of contracting COVID-19, and only 20.2% of the population believed that they had more than 50% risk of contracting the virus (Table 2).

**Table 2:** Responses on vaccine acceptance (N=936) COVID-19: coronavirus disease 2019

Variable	Total: n (%)	Vaccine accepted N=656: n (%)	Vaccine refused N=280: n (%)	p-value
Perceived risk of being infected with COVID-19				<0.001
0-10%	271 (29)	172 (26.2)	99 (35.4)	
10-20%	111 (11.9)	72 (11)	39 (13.9)	
20-30%	142 (15.2)	95 (14.5)	47 (16.8)	
30-40%	121 (12.9)	82 (12.5)	39 (13.9)	
40-50%	102 (10.9)	84 (12.8)	18 (6.4)	
50-60%	91 (9.7)	71 (10.8)	20 (7.1)	
>60%	98 (10.5)	80 (12.2)	18 (6.4)	
I will get vaccinated by a vaccine with 95% effectiveness (for a cost)	722 (77.1)	567 (86.4)	155 (55.4)	<0.001
I will get vaccinated by a vaccine with 50% effectiveness (for a cost)	386 (41.2)	302 (46)	84 (30)	<0.001
If a vaccine with 95% effectiveness is made freely available, I will get vaccinated	799 (85.4)	615 (93.8)	184 (65.7)	<0.001
If a vaccine with 50% effectiveness is provided for free, I will get vaccinated	521 (55.7)	425 (64.8)	96 (34.3)	<0.001
I have confidence in the reliability of media sources regarding COVID-19				<0.001
Strongly disagree	104 (11.1)	35 (5.3)	69 (24.6)	
Disagree	152 (16.2)	96 (14.6)	56 (20)	
Neutral	316 (33.8)	234 (35.7)	82 (29.3)	
Agree	163 (17.4)	127 (19.4)	36 (12.9)	
Strongly agree	201 (21.5)	164 (25)	37 (13.2)	
I get Influenza/flu vaccine/flu shots administered every year	233 (24.9)	177 (27)	56 (20)	0.02
I have trust in the national government in controlling the pandemic				<0.001
Strongly disagree	106 (11.3)	58 (8.8)	48 (17.1)	
Disagree	143 (15.3)	88 (13.4)	55 (19.6)	
Neutral	271 (29)	192 (29.3)	79 (28.2)	
Agree	189 (20.2)	148 (22.6)	41 (14.6)	
Strongly agree	227 (24.3)	170 (25.9)	57 (20.4)	
If I contract COVID-19, it can be debilitating and dangerous to my health				<0.001
Strongly disagree	36 (3.8)	10 (1.5)	26 (9.3)	
Disagree	65 (6.9)	42 (6.4)	23 (8.2)	
Neutral	206 (22)	119 (18.1)	87 (31.1)	
Agree	263 (28.1)	181 (27.6)	82 (29.3)	
Strongly agree	366 (39.1)	304 (46.3)	62 (22.1)	
Source considered the most reliable regarding COVID-19 information				<0.001
Healthcare workers	520 (55.6)	377 (57.5)	143 (51.1)	
Healthcare officials	98 (10.5)	72 (11)	26 (9.3)	
Social media	121 (12.9)	71 (10.8)	50 (17.9)	
Scientific literature/internet	146 (15.6)	117 (17.8)	29 (10.4)	
Other sources	51 (5.4)	19 (2.9)	32 (11.4)	

Overall, 70.1% of the population reported willingness to get the COVID-19 vaccine if available and recommended by healthcare workers. When asked if they would get the vaccine with 95% effectiveness for a cost, 77.1% of our respondents responded in the affirmative, compared to only 41.2% who were willing to get vaccinated with a 50% effective vaccine for a cost. In contrast, 85.4% stated they would get vaccinated with a 95% effective vaccine if freely available. Whereas 55.7% stated that they would get vaccinated with a 50% effective vaccine if freely available. Table 3 presents a univariate analysis of the findings.

**Table 3:** Univariate analysis of the determinants of COVID-19 vaccine acceptance COVID-19: coronavirus disease 2019

Variable	Odds ratio	95% confidence intervals	p-value
Age (years)			
18-19	[reference]		
20-29	2.04	1.48-2.79	<0.001
30-39	1.40	0.86-2.27	0.17
40-49	1.86	0.72-4.78	0.19
50-59	0.78	0.36-1.70	0.54
>60	0.51	0.18-1.45	0.21
Gender			
Female	[reference]		
Male	1.47	1.10-1.97	0.009
Province			
Gilgit-Baltistan	[reference]		
Sindh	1.63	0.47-5.55	0.43
Punjab	0.95	0.28-3.20	0.93
Balochistan	0.91	0.22-3.68	0.90
Khyber Pakhtunkhwa	1.38	0.36-5.18	0.63
Education			
No formal education	[reference]		
Primary school	2.2	0.17-28.13	0.54
Secondary school	3.52	1.33-9.25	0.01
Bachelor/university	2.9	1.21-6.96	0.01
Master/PhD	1.89	0.76-4.68	0.16
Diploma	0.82	0.21-3.21	0.78
Occupation			
Unemployed/retired	[reference]		
Private sector	1.30	0.8-2.11	0.28
Government employee	2.51	0.99-6.35	0.052
Business	0.7	0.33-1.48	0.36
Student	0.77	0.52-1.14	0.20
Income (in Pakistani rupees)			
<10,000	[reference]		
10,000-49,999	1.09	0.5-2.34	0.82
50,000-100,000	1.45	0.65-3.21	0.35
>100,000	1.67	0.77-3.60	0.18
Marital status			
Married	[reference]		
Single	1.09	0.77-1.52	0.61
Divorced	0.25	0.09-0.68	0.007
Occupation			
None of the below	[reference]		
Healthcare worker	2.12	1.33-3.38	0.002
Medical students	1.36	0.99-1.86	0.054
Presence of COVID-19 infection in family or friends			
No	[reference]		
Yes	2.04	1.52-2.73	
Comorbidity			
No	[reference]		
Yes	1.08	0.72-1.63	0.69
Perceived risk of being infected with COVID-19			
0-30%	[reference]		
40-60%	1.68	1.22-2.29	0.001
>60%	2.42	1.41-4.16	0.001
Confidence in the reliability of media sources regarding COVID-19			
No	[reference]		
Yes	2.26	1.66-3.07	<0.001
Influenza/flu vaccine/flu shots administered every year			
No	[reference]		
Yes	1.47	1.05-2.07	0.02
Trust in the national government to control the pandemic			
No	[reference]		
Yes	1.74	1.30-2.33	<0.001
COVID-19 can be debilitating and dangerous to health			
No	[reference]		
Yes	2.67	2-3.58	<0.001
Source considered the most reliable regarding COVID-19 information			
Healthcare workers	[reference]		
Healthcare officials	1.05	0.64-1.71	0.84
Social media	0.53	0.35-0.81	0.003
Scientific literature/internet	1.53	0.97-2.4	0.64
Other sources	0.22	0.12-0.41	<0.001

Over 27.3% of the respondents did not perceive the media to be a reliable source of information regarding COVID-19, whereas 38.9% perceived it to be reliable, and the remaining (33.8%) were neutral to media outlets. When asked to choose what they perceived to be the most reliable source of information regarding COVID-19, 66% of the respondents chose healthcare workers and officials, 15.6% chose scientific literature and the internet, and 12.9% chose social media.

The multivariate analysis identified that first male gender, second healthcare workers and medical students, third respondents whose family or friends had contracted COVID-19, and fourth individuals who trusted media as a reliable source for COVID-19 were more receptive to the vaccine. Participants’ belief that COVID-19 can be debilitating and dangerous to their health was also significantly linked to greater acceptance of the vaccine. The results of the multivariate analysis are depicted in Table 4.

**Table 4:** Multivariate analysis of the determinants of COVID-19 vaccine acceptance COVID-19: coronavirus disease 2019

Variable	Odds ratio (95% confidence intervals)	p-value
Gender		
Female	[reference]	
Male	1.65 (1.2-2.26)	0.002
Occupation		
None of the below	[reference]	
Healthcare worker	2.1 (1.25-3.51)	0.005
Medical students	1.64 (1.16-2.31)	0.004
Presence of COVID-19 infection in family or friends		
No	[reference]	
Yes	2.11 (1.53-2.91)	<0.001
Confidence in the reliability of media sources regarding COVID-19		
No	[reference]	
Yes	2.11 (1.48-3.01)	<0.001
Trust in the national government to control the pandemic		
No	[reference]	
Yes	1.49 (1.06-2.10)	0.02
COVID-19 can be debilitating and dangerous to health		
No	[reference]	
Yes	2.28 (1.68-3.11)	<0.001

## Discussion

The COVID-19 vaccine is being rolled out using a phased approach to immunize individuals in Pakistan to achieve herd immunization. Our study shows that males, healthcare workers, and medical students are more likely to accept the vaccine, whereas the chances of immunizing the masses are higher if the vaccine is given free of cost. Irrespective of vaccination costs, a statistically higher proportion of individuals were keen on getting the vaccine with a 95% efficacy as opposed to those with 50% efficacy rates.

Previously conducted surveys found that adequate knowledge among the general public is a critical determinant of infection prevention and control. In contrast to the more severe second wave of COVID-19 infection in Pakistan, a large cross-sectional survey of 1200 Pakistani residents during the first wave found that 93.3% of the surveyed population had adequate knowledge of COVID-19 precautionary measures [8]. Our study indicates that 70.1% of the population were willing to get the vaccine if it became available and was recommended by a credible source. The finding is aligned with India, another LMIC that has similar literacy rates, and socio-economic conditions to Pakistan. The vaccine acceptance measured among the general public in India was found to be 74% [9]. Among countries with higher incomes and literacy, scientific literature notes lower acceptance rates for the vaccine; with 64.7% in Saudi Arabia [10] and 67% in the United States [7].

We evaluated the importance of factors such as the cost of the vaccine, reported effectiveness, and the duration of protection in determining acceptability in the population. The data showed that if a paid vaccine with 95% and 50% effectiveness became available, only 77.1% and 41.2% of the participants would, respectively, get the vaccine. However, if the two vaccines were made available free of cost, the acceptance would increase to 85.4% and 55.7% concerning vaccines with 95% and 50% effectiveness, respectively. This was in line with the results published by Harapan and colleagues from Indonesia, where 93% and 67% of the participants were willing to receive the vaccine if provided free of cost with a 95% and 50% effectiveness, respectively [6]. Prominent works such as Jeffery and colleagues showed that the differences in acceptance rates ranged from 90% in China to less than 55% in Russia [11].

The myth about the COVID-19 infection and vaccination persists in Pakistan, with religious undertones in the country [12]. Sentiments have been expressed such as the virus being an attack on Islamic nations and the vaccine containing micro-chips allowing governments to gain control of vaccinated individuals through the introduction of fifth-generation technology [12]. Ultimately, this has led to lower community trust, as documented with a 44.5% trust in the government shown in our study. These are worrisome trends since several studies report that populations with higher trust in the national healthcare systems are associated with higher vaccine acceptance and other health services [13,14]. Our data also revealed that 66% of the population considered healthcare professionals and officials as a reliable source of information for COVID-19. Furthermore, a large proportion of vaccine acceptance is seen among healthcare workers and student groups as compared to non-healthcare workers. A systematic analysis by Vasilevska and colleagues found that healthcare workers are at a greater risk of contracting the infection and the central means of protecting themselves, and their families are through vaccination [15]. Healthcare workers may be further engaged by utilizing social media as a means to promote the importance of COVID-19 vaccine acceptance among the community.

Amyn and colleagues evaluated the determinants of COVID-19 vaccine acceptance among the general population in the United States [7]. The general public showed a lower interest in being vaccinated as compared to our study participants; 67% in the United States versus 71% in our study [7]. The study showed that 72% males, 78% adults aged 55 years or above, 81% Asians, and 75% college and/or graduate degree holders were more willing to accept the vaccine as compared to other groups [7]. Even though we had a lower representation of the older age groups in Pakistan, of the 43 older aged individuals who completed the survey, 23 (53.4%) of the individuals were willing to get the COVID-19 vaccine. Similarly, 608 (65%) university students with a bachelor's degree and 199 (21.3%) individuals with a higher-level degree were more willing to partake in the immunization program as opposed to the 21 (2.2%) lesser-educated individuals.

Gul Deniz and colleagues conducted an online survey to ascertain the hesitancy of vaccine administration in the United Kingdom and Turkey [16]. In both countries, around 3% of the individuals presented with no intention of obtaining the COVID-19 vaccine, where 31% of participants in Turkey and 14% in the United Kingdom were unsure about the vaccine [16]. Overall, 54% of the participants in Turkey and 63% in the United Kingdom believed that the outbreak of the novel coronavirus was natural, with no attributable conspiracy theories, finally leading to increased willingness of obtaining the COVID-19 vaccine [16].

Limitations

The present study had certain limitations. Firstly, due to the state-wide lockdowns, the available method to distribute the survey was a multitude of online platforms, employing the non-probability convenient sampling strategy which may not portray an accurate image of the participants in our population. Secondly, groups with limited access to online platforms could not fill our survey, leading to an underrepresentation of individuals from lower socioeconomic classes. Thirdly, around 80% of the participants were aged 30 years or less, and had a bachelor’s degree, leading to an overestimation of vaccine acceptance among the Pakistani population. Notably, the survey was distributed using social media platforms with 76 million Internet users in Pakistan; of which, 63% of users were in the age group of 20 to 25 years, according to the Pakistan Telecommunication Authority [17]. Lastly, this study did not measure the knowledge of COVID-19 among the participants, therefore, we were unable to derive any associations of acceptance with previous knowledge of the disease. However, our study is the first to assess the large-scale acceptance of COVID-19 in Pakistan that strategically evaluates vaccine uptake behaviors tied in with demographic and geographic factors across the country. During the data collection of this survey, several reports were available reporting the efficiencies of vaccines, therefore our findings are based on real-time associations of vaccine trials and administration to acceptance by the Pakistani population.

## Conclusions

This is the first study from Pakistan that depicts the population-level acceptance of the COVID-19 vaccine and the influencing factors. These contributors vary significantly with demographics and geographical disparities. Based on our study findings, the most important determinants of vaccine acceptance were, first, dissemination of credible information, second, source of evidence like healthcare workers or government officials, third, social media influencer channels, and fourth media outlets. The central determinants of vaccine acceptance in Pakistan may be optimized for public health vaccination at the governmental and health sector level to increase compliance of vaccine administration across low- and middle-income countries. The social determinants of vaccine acceptance, demographical and geographical differences, may be utilized to recommend targeted public health strategies and uniform vaccine rollouts for the ongoing COVID-19 pandemic and global outbreaks in the future.
